# COVID-19-associated encephalitis or Creutzfeldt–Jakob disease: a case report

**DOI:** 10.2217/nmt-2021-0025

**Published:** 2021-12-02

**Authors:** Gooya Tayyebi, Seyed Kazem Malakouti, Behnam Shariati, Leila Kamalzadeh

**Affiliations:** ^1^Mental Health Research Center, Psychosocial Health Research Institute, Department of Psychiatry, School of Medicine, Iran University of Medical Sciences, Tehran, Iran

**Keywords:** coronavirus, COVID-19, Creutzfeldt–Jakob disease, encephalitis, prion diseases, SARS-CoV-2

## Abstract

**Background:** Accurate diagnosis and management of patients with rapidly progressive dementia may be challenging during the COVID-19 pandemic, which has negatively influenced the diagnostic performances, medical resource allocation and routine care for all non-COVID-19 diseases. **Case Presentation:** We herein present a case of a 57‐year‐old male with rapidly progressive cognitive decline, headache, diplopia, myalgia, unsteady gait, aggression, depression, insomnia, hallucinations and delusions of persecution. COVID-19-associated encephalitis was briefly considered as a differential diagnosis. However, this hypothesis was rejected upon further investigation. A final diagnosis of sporadic Creutzfeldt–Jakob disease was made. **Conclusion:** A timely and accurate diagnosis of Creutzfeldt–Jakob disease gives patients and their families the chance to receive a good standard of healthcare and avoid extensive evaluations for other conditions.

## Background

Accurate diagnosis and management of patients with rapidly progressive dementia (RPD) may be challenging during the coronavirus disease (COVID-19) pandemic that has negatively influenced the diagnostic performances, medical resource allocation and routine care for all non-COVID-19 diseases [1]. RPDs refer to conditions that develop from the onset of the initial symptoms to dementia in less than 1–2 years, and most of these evolve over weeks to months. Prion diseases are the prototypical causes of RPD; however, many other conditions such as infectious, toxic-metabolic, vascular, metastases/neoplasm-related, autoimmune, iatrogenic, systemic and nonprion neurodegenerative disorders can present as RPD [2]. Notably, COVID-19 can also present as RPD with neurological manifestations including decreased muscle strength, ataxia, dizziness, headache, anosmia, hallucinations, ischemic stroke and intracerebral hemorrhage that develop over days to weeks [3]. Thus, during the COVID-19 outbreak, SARS-CoV-2-associated encephalitis is often the first diagnosis many clinicians take into account while treating a patient with rapidly progressive decline.

Human prion diseases are fatal neurodegenerative diseases caused by the transformation of the cellular prion protein (PrP^C^) into a pathogenic isoform called the prion (prion protein scrapie [PrP^Sc^]) [4]. Prion diseases affect 1–2 cases per million per year, of which 80–95% are sporadic Creutzfeldt–Jacob disease (sCJD), 10–15% are genetic/familial and less than 1% are acquired [4,5]. Although the PrP^C^ conversion is presumed to occur spontaneously in sCJD, with genetic prion diseases, the conformational rearrangement of PrP^C^ is thought to be induced by several mutations in the prion protein gene, *PRNP*. In acquired forms, the accidental transmission of PrP^Sc^ to a person results in misfolding of the endogenous PrP^C^ [6,7]. The mean age of onset of sCJD is approximately 64 years, and the average clinical duration is about 6 months; however, there are several subtypes with slower progression [4]. Cognitive impairment is often the first symptom in sCJD, followed by cerebellar, behavioral and constitutional symptoms such as malaise, dizziness, headache, impaired sleep and weight loss. Higher cortical deficits like aphasia, apraxia, neglect, acalculia, as well as visual disturbances, and oculomotor dysfunction may also develop during the clinical course of the disease [4,8]. Genetic Creutzfeldt–Jacob diseases (gCJDs), which are the most common forms of genetic prion disease, are caused by several *PRNP* mutations [4]. They usually begin between ages 30 and 55, and present with myoclonus, ataxia and other motor abnormalities [4]. The median disease duration of gCJD is about 5 months, similar to that of sCJD [9]. Patients with gCJD generally have neuropathologic, clinical and brain imaging characteristics that are indistinguishable from sCJD [4].

Patients often receive several other diagnoses before their diagnosis of CJD [2]. Given the potential of SARS-CoV-2 to present with rapid cognitive deterioration, it is quite possible that patients with CJD are misdiagnosed as having COVID-19-associated encephalitis. This misdiagnosis could lead to prolonged hospitalization, needless or unfavorable treatments, increased healthcare costs and overuse of healthcare resources [10]. Making an early and correct diagnosis of CJD is crucial for family psychoeducational interventions, optimal symptom management and avoiding the risk of disease transmission [1]. We herein present a case of a 57‐year‐old male who was admitted to our hospital for rapidly progressive cognitive decline during the COVID-19 pandemic.

## Case presentation

In December 2020, at the height of the coronavirus disease (COVID-19) pandemic, a 57-year-old man was referred to our hospital showing symptoms of severe agitation, disorientation and impaired consciousness. Additional information from his family revealed a 5-month history of worsening physical and psychiatric symptoms including headache, diplopia, myalgia, unsteady gait, obsessive thoughts about dirt and disease, fear of death, verbal and physical aggression, depressed mood with frequent crying, psychomotor retardation, insomnia, anorexia, visual and auditory hallucinations, delusions of persecution and cognitive decline.

Past medical history was unremarkable except for benign prostatic hyperplasia, for which he took tamsulosin. No history of substance abuse was reported. His father was diagnosed with schizophrenia at the age of 45 and died of a heart attack at the age of 72. Two siblings were also diagnosed with schizophrenia at the ages of 40 and 42 and were alive now at the ages of 60 and 63, respectively. The patient lived in a village in the Kermanshah province of Iran and made a living through livestock breeding. Over the 3 months prior to his admission to our hospital, the patient was treated with olanzapine, chlordiazepoxide, sertraline, nitroglycerin, analgesics and several herbal medicines, without any significant clinical improvement. One week prior to admission, the patient had undergone a single session of electroconvulsive therapy with the diagnosis of a major depressive episode with catatonia. However, he had progressively deteriorated into disorientation, confusion, difficulty speaking and ataxia.

On examination, the patient was confused, muted, made no eye contact and was unresponsive to commands. He was afebrile, his blood pressure was 170/85 mmHg, heart rate was 88/min, respiratory rate was 19/min and oxygen saturation on room air was 90%. Neurological examination revealed generalized mild muscle rigidity, decreased muscle strength in all four limbs, lower-limb myoclonic jerks and increased deep tendon reflexes. No nuchal rigidity or other meningeal signs were detected. Chest and abdominal examinations revealed no abnormalities.

Based on the presenting symptoms, the possibility of CNS infection (including COVID-19-associated encephalitis), we considered metabolic encephalopathy, autoimmune encephalitis, cerebrovascular accident or toxic insults, Lewy body or Alzheimer’s dementia, vitamin B12 and/or folate deficiency dementia and prion diseases in the initial differential diagnosis.

Baseline laboratory investigations were significant for pancytopenia, elevated C-reactive protein, elevated erythrocyte sedimentation rate, increased creatine phosphokinase and microscopic hematuria. Fasting plasma glucose, hemoglobin A1c, urea, creatinine, serum vitamin B12 and folate levels as well as liver and thyroid function test results were in the normal range. Serum viral markers (hepatitis B surface antigen, hepatitis C antibodies and HIV 1 & 2 antigen and antibody), blood culture, D-dimer, Rapid Plasma Reagin (RPR), Venereal Disease Research Laboratory (VDRL) and heavy metal screening were negative. Initial cerebrospinal fluid (CSF) analysis was normal (Table 1). Chest and brain spiral CT scans were unremarkable. An electrocardiogram and chest x-ray showed no abnormalities.

**Table 1.** Laboratory investigations performed on admission.

**Complete blood count**	WBC: 2.68 × 10^9^/l
	RBC: 2.48 × 1012/l
	Hemoglobin: 11 g/dl
	Hematocrit: 25.8%
	MCV: 80.9 fl
	Platelets: 100 × 103/μl
**ESR**	65 mm/h
**CRP**	25 mg/l
**CSF appearance**	Clear and colorless
**CSF cytology, microbiology and virology**	WBC: 2–3 cells/cmm
	RBC: 5 cells/cmm
	Gram stain: no organism is seen
	Culture: no growth
	COVID-19 PCR: negative
	Herpes simplex virus I and II DNA PCR: negative
	Varicella zoster virus DNA PCR: negative
	Human herpes virus 6 DNA PCR: negative
	Human herpes virus 7 DNA PCR: negative
**CSF analysis**	CSF protein: 32 mg/dl
	CSF glucose: 65 mg/dl
**CSF autoimmune panel**	NMDA-R Ab: negative
	AMPA-R Ab: negative
	DPPX Ab: negative
	CASPR2-IgG: negative
	LGI1-IgG: negative
	GABA-B-R Ab: negative

AMPA-R Ab: α-Amino-3-hydroxy-5-methyl-4-isoxazolepropionic acid receptors antibodies; CASPR2-IgG: Contactin-associated protein-like 2 immunoglobulin G; CRP: C-reactive protein; CSF: Cerebrospinal fluid; DPPX Ab: Dipeptidyl-peptidase-like protein antibodies; ESR: Erythrocyte sedimentation rate; FEU: Fibrinogen equivalent unit; GABA-B-R Ab: Gamma aminobutyric acid receptor, type B antibodies; GGT: Gamma-glutamyl transferase; LGI1-IgG: Leucine-Rich, glioma-inactivated protein 1 immunoglobulin G; NMDA-R Ab: N-methyl-d-aspartate receptor antibody.

**Figure 1.**
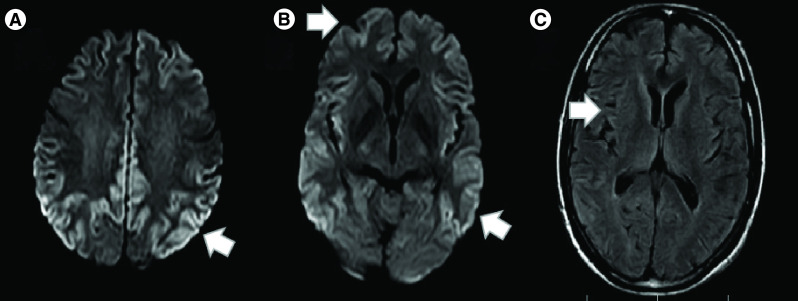
Brain MRI performed on the second day of hospital admission. **(A & B)** Brain MRI, diffusion-weighted imaging and **(C)** fluid-attenuated inversion recovery sequences demonstrated global parenchymal loss as well as bilateral high-intensity areas affecting the frontal, parietal, temporal and occipital cortices (cortical ribbon sign).

The PCR test for COVID-19 with a SARS-CoV-2 nasopharyngeal swab was negative. The cranial MRI performed on the second day of admission (Figure 1) showed global parenchymal loss as well as bilateral cortical ribbon sign on diffusion-weighted imaging (DWI) and fluid-attenuated inversion recovery (FLAIR) sequences.

While waiting for further CSF analysis results, the patient was empirically treated with intravenous acyclovir for viral encephalitis and methylprednisolone for autoimmune encephalitis, which did not result in any clinical improvement. He was also treated with lorazepam for myoclonic jerks with minimal effect.

Results for COVID-19 PCR, herpes simplex virus PCR and antineural autoantibodies from CSF were negative; however, the 14-3-3 protein was found positive in the CSF by Western blot. Electroencephalography was not performed due to the worsening and unstable clinical status.

A final diagnosis of probable sporadic Creutzfeldt–Jakob disease was made, considering the rapid clinical deterioration, CSF findings, negative family history for RPD and no previous iatrogenic exposures.

Other biomarkers of sCJD such as CSF real-time quicking-induced conversion, total tau, PrP^Sc^ and scrapie-associated fibrils were not assessed due to the lack of availability of relevant technologies in Iran.

The patient deteriorated throughout his admission, and he passed away on the 20th day of hospitalization. No postmortem examination was carried out owing to the wishes of the family.

## Discussion

As commonly seen in sCJD [11], this patient had constitutional and behavioral early symptoms. With the progression of the disease, he developed rapid cognitive decline with prominent motor, cerebellar and visual disturbances leading to akinetic mutism and death within approximately 6 months. Given that encephalopathies are quite common in COVID-19, especially in severe cases [12], the rapid decline in the patient’s cognitive status led us to initially suspect COVID-19-associated encephalitis.

Since the emergence of COVID-19, a considerable amount of literature has been published on the neurological manifestations associated with SARS-CoV-2. COVID-19 can mimic many other conditions that involve rapidly progressive cognitive decline [2]. In addition to cognitive impairment, decreased muscle strength, ataxia, dizziness, headache, anosmia, hallucinations, ischemic stroke and intracerebral hemorrhage are the most common neurological manifestations reported in patients with COVID-19 [3]. Neurological manifestations in COVID-19 are likely caused by systematic immune activation as well as direct nervous system involvement [13]. However, this diagnosis was ruled out in our case by subsequent negative nasopharyngeal and CSF COVID-19 PCR.

Besides COVID-19 associated encephalitis, we considered other possible causes of RPD including meningitis or encephalitis of bacterial or viral etiologies, metabolic encephalopathy, autoimmune encephalitis, cerebrovascular accident, toxic encephalopathy, nonprion neurodegenerative disorders and vitamin B12 and/or folate deficiency dementia as our initial differentials. Nevertheless, all the available metabolic parameters were within normal limits. CNS infection and autoimmune encephalitis were ruled out according to CSF analysis and culture results. Cerebrovascular accidents and toxic encephalopathy were also unlikely according to MRI findings and negative heavy metal screening, respectively.

Our case was finally diagnosed with probable sCJD, based on the CDC criteria [14,15]. RPD, myoclonus, extrapyramidal signs, akinetic mutism, positive 14-3-3 CSF assay, hyperintense signal on DWI and FLAIR sequences involving the temporal, parietal and occipital cortex, as well as a global parenchymal loss were suggestive of sCJD [14].

The classic neuroimaging findings associated with sCJD are T2/FLAIR and DWI cortical hyperintense signal changes (cortical ribbon sign) and/or deep gray matter nuclei hyperintensities, with associated restricted diffusion on the apparent diffusion coefficient sequences [16]. The sensitivity (92–96%) and specificity (93–94%) of MRI in the diagnosis of sCJD is greater than almost any other diagnostic test [4]. In sCJD, brain atrophy is typically not an evident early radiological finding by standard visual assessment and appears only in the advanced stages of the disease, such as was the case with the patient described here. As the disease progresses, the cognitive and functional decline is associated with greater atrophy [11]. Despite the high sensitivity and specificity of MRI for sCJD, some other prion and nonprion RPDs present with MRI findings similar to those seen in sCJD and should be considered as differential diagnoses. Wilson’s disease, Bartonella encephalopathy, Wernicke’s encephalopathy, extra pontine myelinolysis and voltage-gated potassium channel-complex antibody-associated dementia may cause reduced diffusion in the cortical and subcortical gray matter [16]. As mentioned earlier, these diseases were excluded in our case based on the results of auxiliary tests. Regarding COVID-19 encephalopathy, neuroimaging findings have been reported to be variable but dominated by intracranial hemorrhages and acute ischemic infarcts. Additional brain imaging patterns in COVID-19 include leukoencephalopathy, cytotoxic lesions of the corpus callosum, olfactory bulb involvement and cranial nerve enhancement, as well as nonspecific cortical hyperintensities on T2/FLAIR with accompanying restricted diffusion that may be caused by systemic toxemia, viremia or hypoxia [17,18].

Unfortunately, due to the lack of availability of *PRNP* genetic testing, we could not definitively rule out the diagnosis of gCJD, as it commonly displays clinical and MRI features resembling those of sCJD [4]. Similar to sCJD, rapidly progressive cognitive decline, cerebellar symptoms, myoclonus, extrapyramidal signs and psychiatric symptoms are quite common in gCJD [9]. Certain mutations such as E200K, the most common *PRNP* mutation worldwide, can have high signals in deep nuclei and particularly cortical ribbon sign on MRI similar to those found in sCJD [4,9]. Moreover, The CSF biomarkers such as 14-3-3, S100β, neuron-specific enolase and total tau are usually elevated in gCJD, but less commonly than they are in sCJD [7]. These proteins are not prion specific, and they are considered to be markers for neuronal injury [4]. Notably, in more than half of the patients with genetic prion disease, no known family history of CJD or neurodegenerative diseases is found. Often; however, there is a family history of neurologic or psychiatric disease that likely has been incorrectly attributed to other causes [19]. As mentioned earlier in this paper, the patient’s father and siblings were diagnosed with schizophrenia; nevertheless, none had a rapidly progressive course of cognitive decline.

Even in the best conditions, early diagnosis and effective care of a patient with sCJD can be challenging [1]. There is often a long delay between the first consultation of a patient with sCJD with a healthcare professional and the time at which an accurate diagnosis is made [20]. Unfortunately, this has been amplified by the disruptions that the COVID-19 pandemic has imposed on medical practice [21]. Our patient had received several psychiatric and medical alternative diagnoses before this admission. This diagnostic delay contributed to costly evaluations for other conditions and hindered the family's ability to focus on end-of-life care.

## Conclusion

Given that the nervous system is a potential target for SARS-CoV-2, it is reasonable to consider COVID-19 associated encephalitis as a differential diagnosis in patients presenting with new‐onset neurological manifestations, with or without the presence of respiratory symptoms. However, it is important that focus on suspected COVID-19 does not lead clinicians to miss the diagnosis of other causes of neurological manifestations, especially rare prion diseases. Although sCJD is invariably fatal and there is currently no accepted treatment available, a timely and accurate diagnosis is crucial since several differential diagnoses are treatable [22]. Therefore, rapid, reliable and relatively noninvasive tests should be used to help early detection of CJD and prevent unnecessary investigations. An early diagnosis will give patients and their families the chance to receive a good standard of healthcare [23,24].

**Summary points**

- COVID-19 can present with neurological manifestations, including rapidly progressive cognitive decline, decreased muscle strength, ataxia, dizziness, headache, anosmia, hallucinations as well as ischemic stroke and intracerebral hemorrhage.
- Given that the nervous system is a potential target for SARS-CoV-2, it is reasonable to consider COVID-19-associated encephalitis as a differential diagnosis in patients presenting with rapidly progressive dementia (RPD).
- However, it is important that focus on suspected COVID-19 does not lead clinicians to miss other causes of RPDs, including rare prion diseases.
- A rapid disease course, presence of myoclonus, detection of 14-3-3 protein in cerebrospinal fluid and MRI findings can help distinguish between sporadic Creutzfeldt–Jakob disease and other causes of RPD.
- Rapid, reliable and relatively noninvasive tests should be used to help early detection of Creutzfeldt–Jakob disease and prevent unnecessary investigations.
